# A Proposal for the Evaluation of HTV Silicone Rubber Composite Insulators

**DOI:** 10.3390/polym13213610

**Published:** 2021-10-20

**Authors:** Christos-Christodoulos A. Kokalis, Vassiliki T. Kontargyri, Ioannis F. Gonos

**Affiliations:** School of Electrical and Computer Engineering, National Technical University of Athens, 9 Iroon Polytechniou Street, 15780 Athens, Greece; vkont@power.ece.ntua.gr (V.T.K.); igonos@cs.ntua.gr (I.F.G.)

**Keywords:** composite insulators, insulator testing, material testing, electrical properties, mechanical properties

## Abstract

This paper describes in detail a step-by-step methodology for obtaining specimens from the housing material (high temperature vulcanized (HTV) silicone rubber with aluminum trihydrate (ATH) filler) of composite insulators (finished products), for five well known and commonly used tests. The aim of the paper is to render practical clarifications and additions to the instructions for five tests on composite insulators provided by international standards. Additionally, the ranges of the results of these tests are presented. More specifically, shore A hardness measurement, tensile strength and elongation at break test, tear strength test, density measurement and inclined plane test were conducted on the housing material of ten, new, unaged medium voltage composite insulators made by six different manufacturers. The results of these tests are presented as a contribution to the existing knowledge and a comparative study of corresponding results of previous investigations is performed. The presented procedures for specimens’ preparation, as well as the results (arithmetical ranges), could be used as guidelines for future testing on the housing material (HTV silicone rubber with ATH filler) of composite insulators, either by researchers and manufacturers, during laboratory testing and material development processes, or by customers (distribution and transmission networks owners), during batch acceptance tests.

## 1. Introduction

Over the last, approximately, five decades, composite insulators have gained more and more ground in the electrical distribution and transmission networks all around the world, replacing the conventional insulators made of porcelain and glass. This wide spread of composite insulators is due to their many advantages compared to conventional insulators, which are mainly their low weight, easy handling and very good performance in heavily polluted environments, thanks to their good hydrophobic property [1,2].

Much research has been done on which material is most suitable as insulating housing for composite insulators’ applications. One of the most commonly used housing materials of composite insulators is high temperature vulcanized (HTV) silicone rubber with aluminum trihydrate, or aluminum hydroxide (ATH) filler. ATH filler is used to increase mechanical properties of the silicone rubber and especially its tracking and erosion resistance [3,4,5,6,7].

The reliable operation of composite insulators during their lifetime in electrical networks depends to a large extent on the condition of their insulating housing material. Therefore, testing of housing material, according to international standards, is considered of great importance [8,9,10].

Five commonly used tests for assuring the quality and reliability of composite insulators’ housing material are: shore A hardness measurement, tensile strength and elongation at break test, tear strength test, density determination and inclined plane test, which are conducted according to the international standards: ISO 48-4 [11], ISO 37 [12], ISO 34-1 [13], ISO 2781 [14] and IEC 60587 [15], respectively. These standards require specimens of specific dimensions in order to conduct the tests, requirement which is not possible to be met on specimens extracted from composite insulators (finished products). In this case, test pieces must be cut from the material of the insulators in an effective and optimized way [8,16,17,18,19].

The results of the above tests on the insulating housing of composite insulators depend on the concentration of ATH in the silicone rubber, as well as on the manufacturing process followed by each manufacturer [5,20,21,22].

In this research, a detailed methodology for obtaining the test pieces from composite insulators (finished products) for the above-mentioned five tests is presented, as well as the results of these five tests on the housing material (HTV silicone rubber with ATH filler) of composite insulators made by different manufacturers. The presented methodologies could contribute to future versions of the ISO/IEC standards of the tests. The presented results are an extension and contribution to the existing knowledge and in combination with previous investigations’ results that are also presented in this article, comprise a useful database for future similar testing on composite insulators.

## 2. Tested Composite Insulators

For the purposes of this article, ten new (unused), commercially available composite insulators were used. These composite insulators were manufactured by six different manufacturers (manufactures, cities, countries) and were intended to be used in outdoor medium voltage applications. The insulating housing material of these insulators was HTV silicone rubber with ATH filler according to their manufacturers’ specifications. In addition, the insulators used for this work belonged to two types: line–post insulators and suspension insulators.

A code name was used for each tested insulator, due to confidential reasons. Code names include a letter from A to F referring to different manufacturers, the number “1” for line-post insulators and the number “2” for suspension insulators. The normal operating voltage level of all tested insulators was as follows: A1, A2, B1, B2, C1, C2, E1 and F1 insulators were manufactured for 24 kV, D1 for 35 kV and D2 for 46 kV.

## 3. Specimens Preparation and Experimental Setups/Procedures for Testing Composite Insulators as Finished Products

In this section, the experimental setups and the specimens’ preparation for some tests on composite insulators are described. The tests conducted in this research work are: shore A hardness measurement (ISO 48-4 [11]), tensile strength and elongation at break test (ISO 37 [12]), tear strength test (ISO 34-1 [13]), density measurement (ISO 2781 [14]) and inclined plane test (IEC 60587 [15]). As mentioned, there are international ISO or IEC standards for each of these tests, which describe the experimental setup and the specimen preparation procedure. Though the aforementioned standards describe in detail the methods for testing material specimens of specific dimensions and shape, there are no certified techniques for extracting specimens from composite insulators (as finished products). These techniques are very important, especially for the customers, in case they want to conduct acceptance tests on a batch of insulators to ensure that they have the specified quality declared by their manufacturers. The procedure for conducting the above-mentioned tests and the possible ways to obtain the test pieces for each test are presented underneath.

### 3.1. Shore A Hardness Measurement

Shore A hardness measurements were conducted to all available composite insulators according to standard ISO 48-4 [11]. Temperature during hardness measurements was in the range of 21 to 22 °C. The test time was set to 3 s as required by the standard for vulcanized rubber [11]. Five measurements were taken on the test pieces, and subsequently, the average value and the standard deviation were calculated.

Regarding the test pieces used for the measurements, the standard [11] requires flat test pieces, which thickness shall be at least 6 mm. The regions of a composite insulator that may be used for this measurement are the sheds of the insulator and the sheath between two sheds. Therefore, there are the following possible specimens that could be used to conduct the measurement on composite insulators:

A. Measurement on the Top Shed

This can be a non-destructive test, as the shed does not need to be cut to make the measurement. The insulator must be placed in such a way that its top shed fits perfectly on a flat and hard surface, for example, on a table.

This procedure deviates from the requirements of the standard in two points [11]. Firstly, in most cases, the thickness of the sheds of a composite insulator is less than 6 mm. The second deviation from the standard requirements is that the surface to be measured is not flat, but it has a small inclination, due to manufacturing design [11]. Additionally, the measurement with this setup may be impossible in some cases of composite insulators, in which the upper metallic end-fitting of the insulator will not allow the hardness tester to fit properly on the surface of the top shed.

B. Measurement on a Half Shed

Another procedure for measuring the hardness on composite insulators is to cut the half of one of the sheds of the insulator and conduct the measurement on it. This is a destructive test because after cutting the shed, this insulator can no longer be used. After cutting the half shed it must be placed on a flat and hard surface in order to make the measurement. In this case, the above-mentioned deviations from the standard requirements [11], regarding the thickness and the flatness of the specimen, continue to apply exactly as before.

This procedure has the advantage that the measurement can be much more accurate than the previous one, because the hardness tester can fit better on the specimen. On the other hand, the disadvantage of this option is that the test in this case is destructive, as was explained before.

C. Measurement on Two Layered Half Sheds

In order to eliminate the aforementioned deviation from the standard regarding the 6 mm thickness of the specimen, two half sheds can be layered and placed on a flat and hard surface to make the measurement on the top half shed [8,11]. These half sheds can be layered in the same direction or in the opposite direction.

In this way, the thickness of the specimen is bigger than 6 mm, which is also the advantage of both these setups. Though the previous advantage, the setup of the two layered half sheds in the same direction still has the disadvantage of the inclined surface of the upper shed under measurement, which is now more intense from the previous cases. As a result, the measurement accuracy is low. 

On the contrary, the setup of the two layered half sheds in the opposite direction uses the two inclined surfaces of the two sheds and creates a flat surface where the hardness tester can fit properly and increase the accuracy of the measurement.

D. Measurement on the Sheath between Two Sheds

The last setup that could be used to conduct the hardness measurement is to cut two consecutive sheds of the insulator and make the measurement on the remaining insulating housing material, the so-called sheath of the insulator.

This setup also has the two above-mentioned deviations from the standard [11]. The thickness of the sheath of the insulator is usually less than 6 mm and the surface of the sheath is circular and not flat, as the standard requires [11]. Therefore, the measurement with this setup also has low accuracy.

As far as the digital hardness meter is concerned, this device can be used by two ways to conduct the shore A hardness measurement on a composite insulator. These ways are as follows:

E. Hand-Held

The most common and effective way to use the digital shore A hardness tester (durometer) in order to make measurements on the housing material of a composite insulator, is to use it as a hand-held (portable) device. The advantage of this way is that the inclination of the durometer can be adjusted by the user in such a way that the durometer can fit properly on the measuring surface. In this way, the measurement can be conducted more accurately.

F. Mounted on a Stand

The second way to use a durometer is to mount it on a stand. This way can give more accurate results only when the measurement is conducted on specimens which are produced according to the instructions of the standard ISO 48-4 [11]. 

In the case of specimens extracted from a composite insulator (finished product), the hardness tester should not be mounted on a stand, because in this case, the inclination of the durometer cannot be properly adjusted on the measuring surface of the insulator. As a result, the measurements will be inaccurate.

In this article, the shore A hardness measurements were conducted firstly on one half shed and secondly on the top shed of two half sheds layered in opposite direction. Furthermore, all measurements were performed using a Bareiss HPE II digital hardness tester (durometer) as hand-held device. These experimental setups are recommended to be used in this kind of measurements, as they are easier and more accurate than the rest.

### 3.2. Tensile Strength and Elongation at Break Test

Tensile strength and elongation at break (or maximum elongation, or ultimate elongation) tests were conducted to all insulators included in this test program. These tests were conducted according to the guidelines of ISO 37 standard [12]. The temperature of the laboratory during these tests was in the range of 21–22 °C.

According to the ISO 37 standard, the test pieces for this test can have two shapes. They can be either “dumb-bell” or “ring” test pieces [12]. The most common shape of the test pieces for this test, when referring to composite insulators, is the dumb-bell shaped test pieces [18,20,22,23].

When conducting the tensile strength and elongation at break test, great importance should be given at the type of the dumb-bell shaped test pieces. The ISO 37 standard specifies five types of dumb-bell shaped specimens. These types are 1, 2, 3, 4 and 1A, which have different dimensions. According to the standard, results obtained from different types of test pieces are not intended to be comparable [12].

This standard describes the preparation of test pieces with specific dimensions that must be specifically constructed for this test [12]. Therefore, when this test must be applied to the material of composite insulators (as finished products), some deviations from the standard seem to be inevitable. 

The only parts of a composite insulator (finished product) that could be used for this test are the sheds of the insulator. A cutting die (with specific dimensions depending on the selected dumb-bell type test piece) is used to cut the test pieces from the sheds of the insulator. ISO 37 requires 2.0 ± 0.2 mm thickness for dumb-bell type 1, 2, 3, 1A test pieces and 1.0 ± 0.1 mm for type 4 test pieces [12]. The thickness of the sheds of composite insulators, as finished products, usually exceeds the thickness of 2 mm, and therefore this deviation from the standard [12] is inevitable.

**Figure 1:**
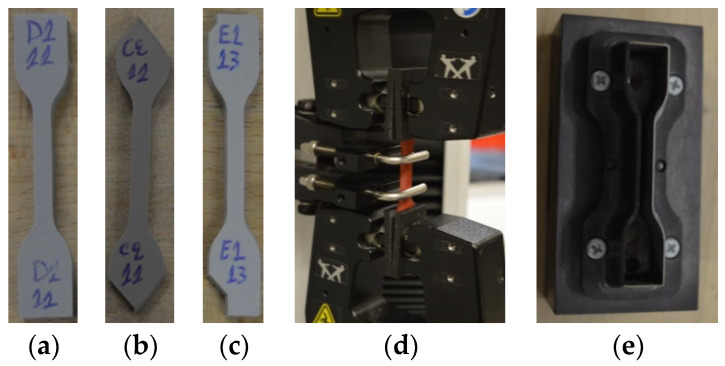
(**a**) Complete dumb-bell test piece, (**b**,**c**) examples of incomplete dumb-bell test pieces due to small size of composite insulators sheds, (**d**) symmetrical fixation of an incomplete (at the edges) test piece on the tensile testing machine, (**e**) the type 2 dumb-bell cutting die that was used in this research.

Furthermore, a second deviation from the standard refers to the shape of the dumb-bell test pieces. Many times, the material of sheds of composite insulators is not enough to cut from them a complete dumb-bell test piece, in the way that it is described in the standard [12] and is illustrated in Figure 1a. In these cases, the tests pieces could be incomplete at their edges due to lack of material (Figure 1b,c). Experience has shown that if the edges of the test pieces are gripped symmetrically to the arms of the tensile testing machine, then the tensile force is applied uniformly to the test piece and the result of the test is accurate (Figure 1d).

In this research, a type 2 dumb-bell cutting die was used for the preparation of the test pieces, as shown in Figure 1e. Five test pieces were cut from the sheds of each insulator. Thickness was measured at the middle point of each test piece and then the average value and the corresponding standard deviation were calculated for each pack of five test pieces. Subsequently, the test was conducted to each test piece and the average value and the standard deviation of both tensile strength and elongation at break were calculated for each pack of five values. The tests were conducted using an Instron 3344 tensile testing machine.

### 3.3. Tear Strength Test

The test for determining the tear strength of the insulating housing material was conducted to all available composite insulators according to the standard ISO 34-1 [13]. During these tests the temperature of the laboratory was at the range of 21 to 22 °C.

According to the standard instructions there are three type of test pieces, which can be used for this test. These are: the “trouser” test piece, the “angle” test piece and the “crescent” test piece. When the crescent test piece is used to conduct this test, there is also a requirement for a nick of depth 1.0 ± 0.2 mm at the middle of the test piece, which is used to initiate the tearing of the material during the test. This nick must be created with great precision to all specimens so that the results are comparable. Angle test piece can be used with or without a nick and trouser test piece does not have requirement for a nick [13]. There is research in the international literature that refers to tear strength results without mentioning the type of the test pieces that were used for the test [4,19,24]. The determination of the used test piece in every tear strength test report is very important so that the results can be comparable with other results of similar investigations.

A cutting die is used for each of the aforementioned cases of test pieces. Similarly to the previous two tests (hardness measurement and tensile strength test), there is a requirement in the standard ISO 34-1 about the test pieces, which specifies specimens with specific dimensions and thickness [13]. The thickness of the test pieces must be 2.0 ± 0.2 mm, which is impossible for the most cases of test pieces extracted from composite insulators (finished products). The only parts of a composite insulator that could be used, as specimens, for this test are the sheds of the insulator, which are usually thicker than 2 mm. Therefore, this is an inevitable deviation from the standard requirements [13].

**Figure 2:**
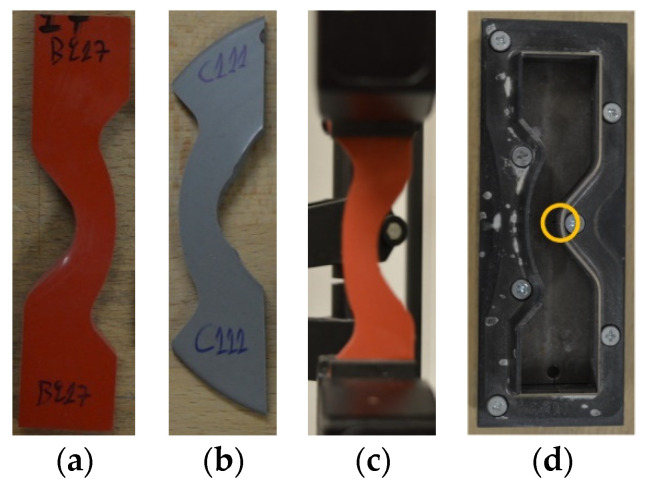
(**a**) Complete crescent test piece, (**b**) example of incomplete crescent test piece due to small size of composite insulator’s shed, (**c**) correct fixation on the tensile testing machine of an incomplete (at the edges) test piece, (**d**) the crescent cutting die used in this investigation and the metallic ledge (in orange circle), which is used to create the required, by the standard ISO 34-1 [13], nick on the test piece.

The second unavoidable deviation from the standard refers to the dimensions of the test pieces. Many times, when this test must be conducted on composite insulators (as finished products), the material of the shed may be less than the specified from the standard (Figure 2a) [13] and therefore, the test piece may be incomplete at the edges (Figure 2b). Nonetheless, experience on this test has revealed that if the test piece can be grabbed appropriately by the arms of the tensile testing machine (Figure 2c), then the result will be accurate.

In this investigation, a crescent test piece cutting die was used and five test pieces from each insulator were cut. The cutting die can be seen in Figure 2d. In addition, in Figure 2d, the metallic ledge on the cutting die marked with an orange circle is used to create the required, by the standard ISO 34-1, nick on the test piece [13]. The test pieces were obtained from the sheds of the insulators. Thickness of each test piece was measured at the point of nick, and the average value and standard deviation were calculated for each pack of five test pieces. After conducting the test, the average value and the standard deviation were calculated for each pack of five measurements. An Instron 3344 tensile testing machine was used for these tests.

### 3.4. Density Measurement

Density measurements were conducted to the insulating housing material of all composite insulators included in this research according to the guidelines of ISO 2781 standard. This standard describes two methods to measure the density of rubber materials, Method A and Method B [14].

When conducting the density measurement with method A, a laboratory analytical balance with ±1 mg accuracy, a balance pan straddle, a beaker of 250 cm^3^ capacity and some distilled water are required according to standard ISO 2781. The test piece shall be a piece of rubber with surfaces out of dust and cracks, which also should be smoothed. Mass of specimen should be at least 2.5 g [14]. Method A is the most commonly used method for density measurements on rubbers.

On the other hand, method B should only be used in cases that there is a suspicion of enclosed air in the core of the rubber material. In method B, a laboratory analytical balance with ±1 mg accuracy, a density bottle and some distilled water are required. The test piece in method B consists of some small pieces, which should weigh at least 2.5 g and which dimensions should be within 4 × 4 × 6 mm [14]. The edges of these small pieces should be smooth. The method B procedure is more tedious than the one of method A [25].

**Figure 3:**
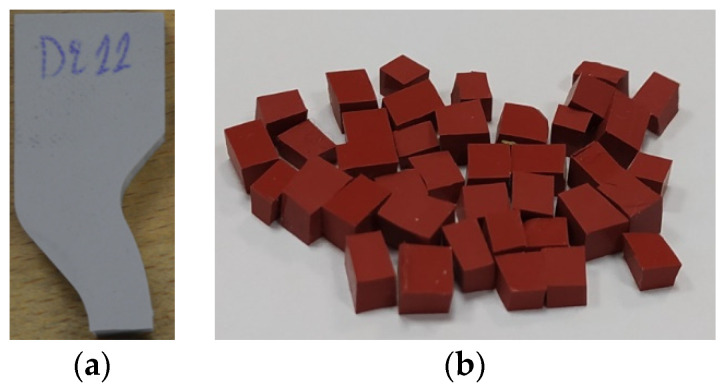
Test pieces for the density measurement from the sheds of composite insulators for (**a**) method A and (**b**) method B of the standard ISO 2781 [14].

The standard ISO 2781 [14] does not have specific instructions on how to conduct the test on composite insulators (as finished products). In the case of composite insulators, the test pieces can be cut from the sheds or from the sheath of the insulators [8]. In Figure 3a, a test piece for method A and in Figure 3b, a test piece (consisting of many small pieces) for method B are illustrated. These test pieces were extracted from the shed of composite insulators.

The main source of error in the density measurement of composite insulators’ housing material is the air bubbles that are created and adhered to the surface of the test piece when it is immersed into the distilled water. It is recommended to use a surface-active material like a detergent in order to reduce the surface tension of the test piece and as a result, to reduce the air bubbles [2,14,25].

In this paper, density measurements were conducted according to the aforementioned method A. Half of the tear strength tests’ samples were used as test pieces (Figure 3a) in order to ensure some uniformity in the shape and mass of the test pieces. These test pieces were obtained after conducting the tear strength tests. This practice (of acquiring the test pieces after the end of the tear strength tests) can be adopted when there is limited housing material of composite insulators. Three density measurements were conducted to each of the available insulators and then the average value and the standard deviation were calculated. During measurements, the temperature of the laboratory and of the distilled water was at 21 °C. Triton X-100 was used as detergent to reduce the air bubbles adhered on the test pieces when immersed in the distilled water. All density measurements were conducted using a Kern PLT 2000 3DM laboratory analytical balance. In addition, a pan straddle and a beaker were used.

### 3.5. Inclined Plane Test

The inclined plane test was conducted to samples extracted from all available insulators. Standard IEC 60587 describes the procedure to perform this test [15]. Five rectangular samples with specific dimensions (50 mm (width) × 120 mm (length)) and thickness (6 mm) are required to be tested according to the standard [15]. More specifically, there are two methods to conduct this test: according to method 1, a constant tracking ac voltage (2.5 kV, 3.5 kV or 4.5 kV) is applied to the specimens for 6 h. On the other hand, according to method 2, a voltage that increases during the test-time is applied to the samples. When the inclined plane test must be conducted on composite insulators, method 1 is recommended by the technical report IEC 62039 [26].

In this test, the samples are placed inclined with the test surface looking downwards at an angle of 45° ± 2°. Two metallic electrodes, of different design and shape, are positioned at the top and bottom edge of the specimen with a distance of 50 ± 0.5 mm between them. The selected voltage is applied between these two electrodes. During the test, a contaminant liquid flows from the top electrode to the bottom one through the testing surface [15]. This contaminant is used, as a wetting agent, in order to eliminate the hydrophobic properties of the specimen’s surface [8].

There are two criteria related to the way that this test is completed (failed). One of them should be chosen as end point criterion for the test result. According to criterion A, there should be no leakage current more than 60 mA for more than 2 s, no hole shall be created on the sample due to intensive erosion and the specimen must not be burned. In criterion B, it is described that the end of the test comes when: the conducting path on the surface of the specimen, which is created during the test, reaches the mid-point of the distance between the two metallic electrodes used for the application of the ac voltage, or a hole on the sample is created, or the specimen ignites. If the 6-h duration of the test is completed and the selected criterion is not met in any of the five required samples, then the test is successful; otherwise, even if only one sample fails, the test result is not successful [15]. According to the technical report IEC 62039, criterion A is recommended as end point criterion for this test, when the test is conducted on composite insulators [26].

The parts of a composite insulator, which could be used as test pieces for the inclined plane test, are the sheds [17] and the sheath of the insulator [8,16]. In this research, all samples were cut from the sheds of the available composite insulators. Experience has shown that the half of the shed is enough to be used as test piece for the inclined plane test. As a result of using the half sheds as specimens for the test, there are two unavoidable deviations from the procedure of standard IEC 60587 [15] regarding the samples for the test.

The first deviation relates to the thickness of the specimens. Standard IEC 60587 requires specimens with 6 mm thickness [15], but in the case of specimens extracted from composite insulators (as finished products) the thickness of the specimens depends on the thickness of the sheds, which is commonly less than 6 mm. 

**Figure 4:**
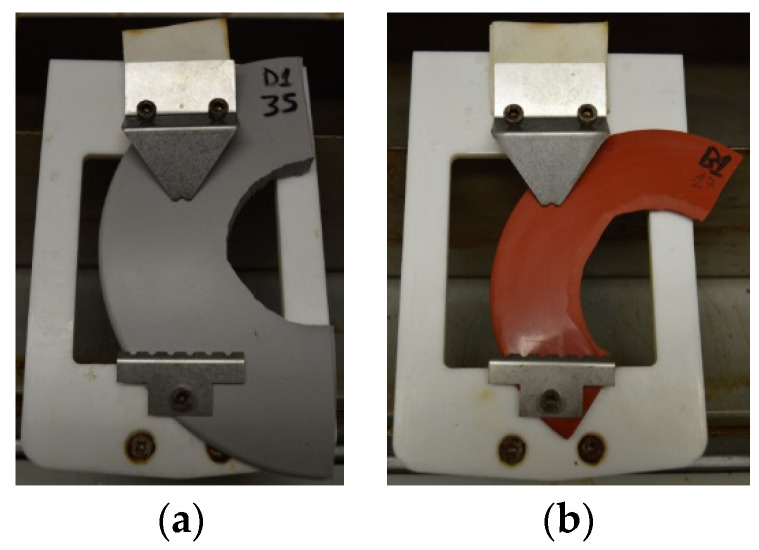
A big (**a**) and a small (**b**) half shed of different composite insulators used as specimens for the inclined plane test.

Additionally, the shape and the dimensions of the samples extracted from composite insulators cannot be as the required from the standard [15]. The shape of the specimens is a semicircle instead of a rectangle and the dimensions are determined by the size of the sheds of each composite insulator. In Figure 4a, a half shed cut from an insulator with relatively big sheds is illustrated. On the contrary, in Figure 4b, a relatively small half shed is presented.

In this research, five samples (half sheds) were used from every available composite insulator, as mentioned before. The temperature of the laboratory during the tests was in the range of 21 to 22 °C. Tests were conducted according to method 1 of the standard IEC 60587 with a constant ac voltage of 4.5 kV, which is the worst case of voltage level mentioned in the standard [15]. Test duration was set at 6 h and criterion A was used as end-point criterion. After the test, maximum depth erosion of each sample was measured and the average value and the corresponding standard deviation of every pack of five samples (five samples per insulator) were calculated. The composition of the contaminant solution included: (a) 0.1 ± 0.002% by mass of ammonium chloride (NH_4_Cl) and (b) 0.02 ± 0.002% by mass of Triton X-100 (as wetting agent) in distilled water. The flow rate of the contaminant was adjusted at 0.6 mL/min. It is worthy to mention that, after each test, both electrodes of each sample were replaced by new ones of the same manufacturer. In this way, the results would not be affected, firstly, by the bad condition of the already used electrodes and secondly, by the different manufacturing process of different manufacturers’ electrodes. A device for inclined plane test made by Alpha Tec Medientechnik GmbH was used to conduct all the inclined plane tests.

## 4. Results

In this section, the results of the five aforementioned tests are presented. What is more, as these insulators (and their materials) have successfully passed all design, type, routine and acceptance ISO–IEC tests according to their manufacturers’ specifications, and as these insulators are made by six different manufacturers from around the world, the range of the results for each test, which is given in this section, could be used as an addition to the already existing knowledge about the insulating material (HTV silicone rubber with ATH filler) of composite insulators. More specifically, the results presented in this section are useful for verifying composite insulators’ housing material.

### 4.1. Shore A Hardness Measurement

**Figure 5:**
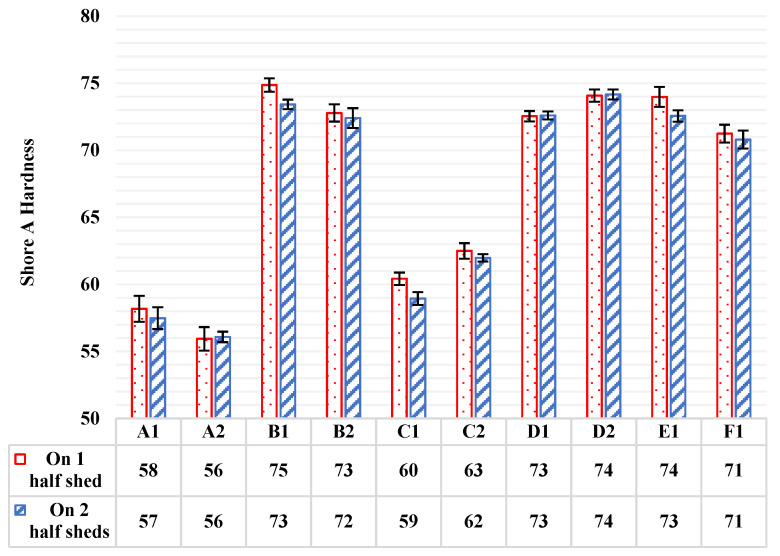
Average values (columns) and standard deviations (error bars) of shore A hardness measurements on one half shed and on two half sheds, made of HTV silicone rubber with ATH filler, of all available composite insulators.

Shore A hardness was measured on HTV silicone rubber with ATH filler material of all available composite insulators, according to the ISO 48-4 standard [11]. As mentioned in Section 3.1 of this paper, five measurements were taken and then the average value and the standard deviation of them were calculated. Measurements were taken firstly on one half shed of each insulator and subsequently on the top of two half sheds, layered in the opposite direction. The average values and the standard deviations of these measurements are illustrated in Figure 5 by columns and by error bars on the top of the columns, respectively.

In Figure 5, it can be observed that the range of shore A hardness measurements on the material (HTV silicone rubber with ATH filler) of composite insulators that are available in the global market (like the tested ones in this research) is approximately in the range of 56 to 75 shore A hardness units, when measuring on one cut half shed or on the top of two half sheds, layered in opposite direction. We can see in Figure 5 that there are some quite significant deviations among the results of different manufacturers. This could be due to the different blends of the materials (fillers) that each manufacturer uses. It has been proven from previous research works that the shore A hardness measurements of the housing material of composite insulators are affected in a proportional way with the concentration of the ATH filler [20,21,27]. In this manner, it seems that B1, B2, D1, D2, E1 and F1 insulators might have higher ATH filler concentrations than A1, A2, C1 and C2 insulators. Additionally, there are some small deviations between the measurements of the two different insulator types (suspension–line post) of each manufacturer. These deviations might be due to the different manufacturing processes of the two types of insulators.

Two series of measurements were taken, on one and on two sheds, in order to make comparisons between them. The main difference of these two experimental setups is that when measuring on one shed, there is usually deviation from the guidelines of standard ISO 48-4 [11], regarding the 6 mm thickness of the sample. The thickness of sheds of common composite insulators that are available in the global market is usually less than 6 mm. On the contrary, measurement on the top of two half sheds, layered in the opposite direction, meets the requirement for the 6 mm thickness of the specimen. In Figure 5, it is observed that the shore A hardness measurement on one half shed gives slightly higher values than measuring on two half sheds. However, this difference is small. A correction factor (k) was calculated, which could be used when measuring on one half shed (with the 6 mm thickness deviation from the standard [11]) to convert this value to the measurement on two half sheds (where the 6 mm thickness requirement is met). In this way, the shore A hardness measurement could be non-destructive for the composite insulators, as the measurement could be conducted on the top shed. Correction factor (*k*), according to Equation (1), average value and standard deviation were calculated and are presented in Table 1. As a result, the correction factor is *k* = 0.99. The 0.01 standard deviation value indicates that the calculated k values for all insulators have small deviation from the calculated average value, and therefore the correction factor is accurate enough.

(1) $$k=\frac{SHA_{2}}{SHA_{1}}$$

where:

*SHA*_2_: is the average shore A hardness measurement on the top of two half sheds, layered in opposite direction.
*SHA*_1_: is the average shore A hardness measurement on one half shed.

Then, Equation (2) could be used to convert the measurement on one half shed to the measurement on two half sheds.

(2) M2=k×M1

where:

*M*2: is the shore A hardness on two half sheds (shore A hardness units).
*M*1: is the shore A hardness measurement on one half shed (shore A hardness units).
*k*: is the correction factor (*k* = 0.99).

Alternatively, due to the small effect of *k* factor in Equation (2), this factor could be included as an uncertainty parameter during the calculation of the total *M*1 measurement uncertainty. 

### 4.2. Tensile Strength and Elongation at Break Test

Tensile strength and elongation at break (or maximum elongation, or ultimate elongation) tests were conducted on five samples from each composite insulator, according to ISO 37 standard [12]. The dimensions of the specimens were determined by the type 2 dumb-bell cutting die that was used. 

The tensile strength values were calculated using Equation (3), while Equation (4) was used for the calculations of elongation at break (or maximum elongation, or ultimate elongation) values, according to the ISO 37 standard [12].

(3) $$TS=\frac{F_{max}}{W\times d}$$

where:

*TS*: is the tensile strength (N/mm^2^).
*F_max_*: is the maximum tensile force recorded at the moment that the specimen breaks (N).
*W*: is the width of narrow portion of the test sample (mm) and it is specified in the standard ISO 37 [12]. For type 2 dumb-bell test piece that is used in this research, this value is equal to 4 mm.
*d*: is the thickness of the sample (mm) (ISO 37 requires 2.0 ± 0.2 mm thickness for type 2 dumb-bell test pieces [12]).

(4) $$EB=\frac{100\times \left(L_{B\;}-L_{0}\right)}{L_{0}}$$

where:

*EB*: is the elongation at break (%).
*L_B_*: is the test length of the specimen at the moment of rupture (mm).
*L*_0_: is the initial test length of the specimen before the test (mm).

**Figure 6:**
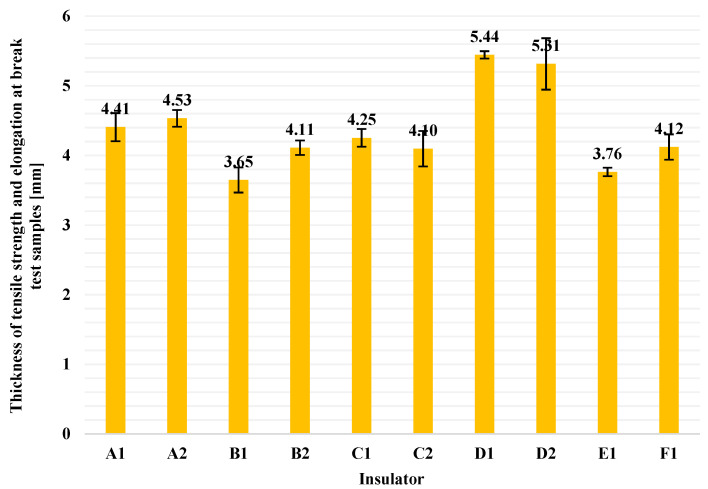
Average thickness values of each pack of five samples (columns) and the corresponding standard deviations (error bars) used for tensile strength and elongation at break test.

The average thickness values of each pack of five samples (columns) and the corresponding standard deviations (error bars) are illustrated in Figure 6. It is observed that the thickness of the tested samples differ from the 2.0 ± 0.2 mm thickness that ISO 37 requires [12]. The deviation regarding the thickness of the specimens seems to be overcome, in some way, since Equation (3) is used for the calculations. More specifically, in Equation (3), there are three factors. *W* factor is determined by the type of dumb-bell test piece that is used and, in this research, *W* is always equal to 4 mm. The other two factors, *F_max_* and *d*, are proportional quantities (i.e., as the thickness of the sample increases, the required tensile force to which the sample breaks will be increased too and vice versa). Therefore, it seems that the thickness of the specimen does not directly affect the result and in this way the results of this test are accurate enough, even though the thickness of the samples is somewhat bigger than the required from ISO 37 standard (2 mm) [12]. From Figure 6, it can be also observed that there were differences among the sample thickness of different manufacturers. This is because of the different manufacturing design of the sheds of different manufacturers.

**Figure 7:**
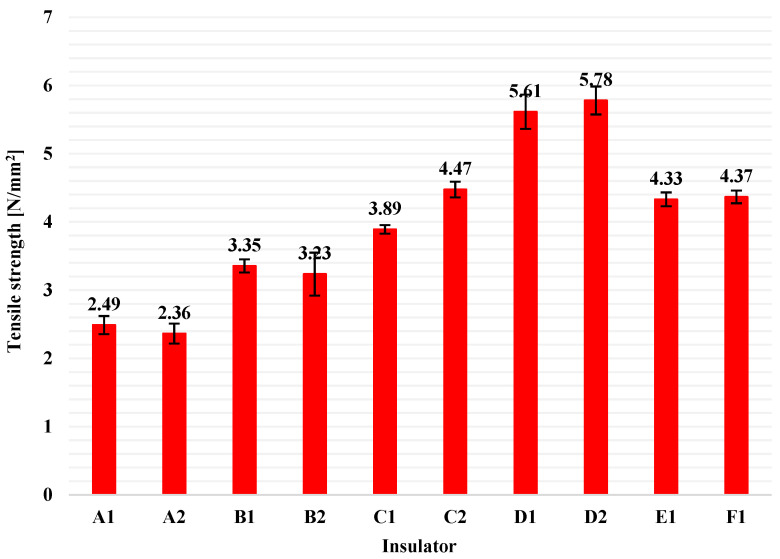
Tensile strength average values (columns) and the corresponding standard deviations (error bars) for the HTV silicone rubber with ATH filler material of all available composite insulators.

**Figure 8:**
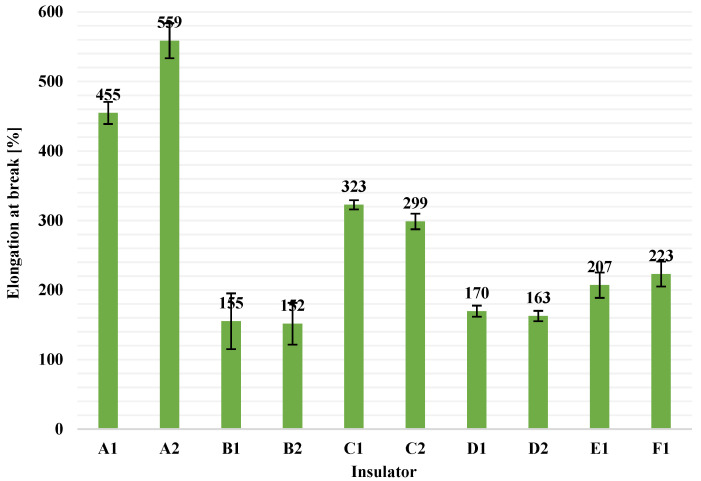
Average values (columns) of elongation at break (or maximum elongation, or ultimate elongation) and the corresponding standard deviations (error bars) for the HTV silicone rubber with ATH filler material of all available composite insulators.

After the test, the average value and the standard deviation of each pack of five values for tensile strength and for elongation at break were calculated. In Figure 7, the average tensile strength values and the corresponding standard deviations, for all available insulators in this study, are illustrated as columns and error bars, respectively. Similarly, average values of elongation at break and the corresponding standard deviations for all insulators are presented in Figure 8.

From Figure 7 and Figure 8, it can be seen that the range of tensile strength values and of values of elongation at break on the material (HTV silicone rubber with ATH filler) of composite insulators that are available in the global market (like the tested ones in this research) is approximately in the range of 2.4 to 6.0 N/mm^2^ (for tensile strength) and 150 to 560% (for elongation at break), when type 2 dumb-bell test pieces are used. In addition, from Figure 7 and Figure 8 it can be observed that there are some considerable deviations on the results, especially among insulators made by different manufacturer. This is most likely due to the different material blends and manufacturing processes that each manufacturer uses. According to the literature, as high as possible tensile strength of the housing material is required, as this prevents damage from wind, sand and bird pecking when the insulators are installed in the electrical network [28]. 

### 4.3. Tear Strength Test

Tear strength test was conducted to the HTV silicone rubber with ATH filler housing material of all available insulators, according to ISO 34-1 standard [13]. Five crescent-type test pieces from each insulator were tested. Equation (5) was used for the calculations of tear strength values, according to standard ISO 34-1 [13].

(5) $$TS=\frac{F_{max}}{t}$$

where:

*TS*: is the tear strength (N/mm).
*F_max_*: is the maximum tensile force till the tearing of the sample (N).
*t*: is the thickness of the specimen at the point where the tear occurred (mm) (ISO 34-1 requires 2.0 ± 0.2 mm thickness [13]).

**Figure 9:**
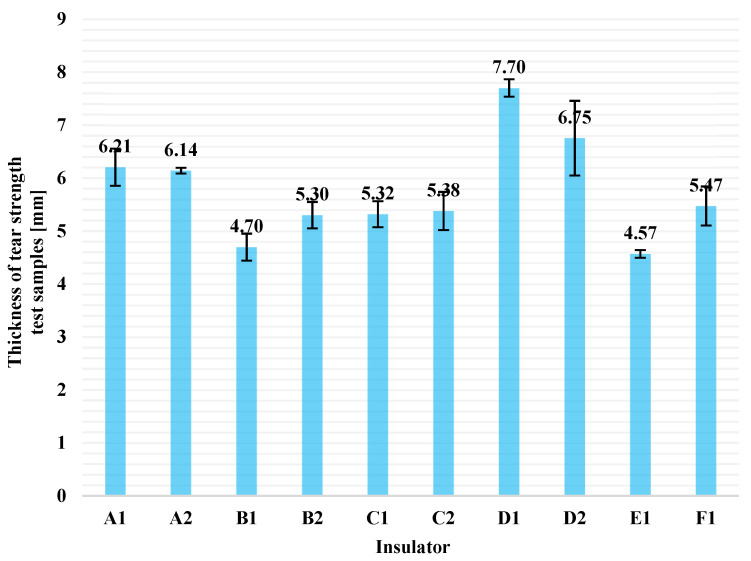
Average thickness values of each pack of five samples (columns) and the corresponding standard deviations (error bars) used for tear strength test.

Average thickness values of each pack of five test pieces (columns) and the corresponding standard deviations (error bars) are illustrated in Figure 9. The deviation mentioned in Section 3.3, regarding the thickness of the specimens, seems to be overcome, in some way, since Equation (5) is used for the calculations. In Equation (5), *F_max_* factor and *t* factor, are proportional quantities (i.e., the thicker the sample, the greater the maximum tensile force required to tear the sample and vice versa). Thus, it seems that thickness of the specimen does not directly affect the result. For this reason, the results of this test are accurate enough, even though the samples are somewhat thicker than the required 2.0 ± 0.2 mm from ISO 34-1 standard [13]. From Figure 9, it is obvious that the thickness of all samples were greater than 2.0 ± 0.2 mm. This is because the samples were cut from the sheds of real composite insulators (finished products) and the thickness of these sheds is determined by the construction design of each manufacturer.

**Figure 10:**
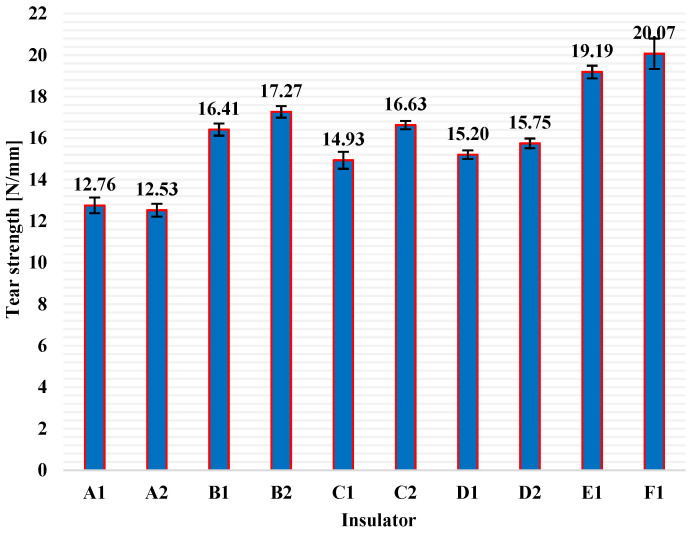
Tear strength average values (columns) and the corresponding standard deviations (error bars) for the HTV silicone rubber with ATH filler material of all available composite insulators.

Tear strength test was conducted to all test pieces obtained from all available insulators and the average values of tear strength (columns), and the corresponding standard deviations (error bars), for each pack of five test pieces, are presented in Figure 10. In Figure 10, it is obvious that the range of tear strength values for the housing material (HTV silicone rubber with ATH filler) of composite insulators available worldwide (like the tested ones) is approximately between 12.5 and 20.0 N/mm, when crescent-type test pieces are used. What is more, in Figure 10 there are some remarkable deviations in the tear strength values of insulators of different manufacturers. This is probably due to the different material blends and manufacturing processes used by each manufacturer. According to previous research works, as high as possible tear strength of the housing material is required, as this prevents damage from careless handling, strong wind and bird pecking when the insulators are going to be installed or are already installed in the electrical network [29,30,31].

### 4.4. Density Measurement

Density measurements were conducted to all available insulators of this research according to method A of ISO 2781 standard [14]. Three measurements were taken from the housing material of each insulator, and consequently, the average and standard deviation values were calculated for every pack of three measurements. Density values were calculated using Equation (6) according to ISO 2781 [14].

(6) $$p=p_{w}\times \frac{m_{1}}{m_{1}-m_{2}}$$

where:

*p*: is the density (g/cm^3^).
*p_w_*: is the density of distilled water (g/cm^3^). All measurements in this research were conducted at 21 °C, and therefore *p_w_* = 0.997989 g/cm^3^ was used in all calculations [32].
*m*_1_: mass of the test piece by weighing it in air (g).
*m*_2_: mass of the test piece by weighing it in distilled water (g).

**Figure 11:**
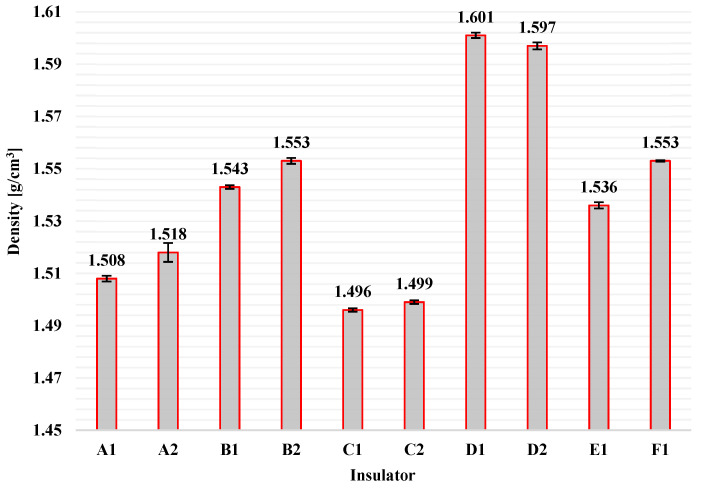
Average values of density measurements (columns) and the corresponding standard deviations (error bars) for the HTV silicone rubber with ATH filler material of all available composite insulators.

The results of density measurements for each of the available insulators are illustrated in Figure 11 by columns. Additionally, the corresponding standard deviations are shown in Figure 11 by error bars.

It can be concluded from Figure 11 that the range of density of the insulating housing material (HTV silicone rubber with ATH filler) of composite insulators that are available on the global market (such as those used in this research) is approximately between 1.5 g/cm^3^ and 1.6 g/cm^3^. It has been found that the density measurement of the housing material of composite insulators can be a first indicator of the material composition, i.e., it can be verified whether the material (silicone rubber) contains some fillers or not [25,33]. Additionally, density measurement can be an indicator for the manufacturing procedure that is used to vulcanise the silicone rubber. According to the literature, the density of HTV silicone rubber is greater than 1.5 g/cm^3^, while RTV (Room Temperature Vulcanized) silicone rubber and LSR (Liquid Silicone Rubber) have lower densities [2]. In Figure 11 there are some noticeable deviations on the density measurements in particular among insulators manufactured by different manufacturers. This might be because of the different concentrations of the ATH filler in the silicone rubber or due to different manufacturing processes. However, all densities are greater or equal to 1.5 g/cm^3^, which means that the housing material is indeed HTV silicone rubber, as it is mentioned in the specifications of the manufacturers.

### 4.5. Inclined Plane Test

Inclined plane test was conducted to all composite insulators included in this research according to method A of IEC 60587 standard [15], where a constant ac voltage of 4.5 kV was applied to the specimens constantly for 6 h, while the flow rate of the contaminant liquid was set to 0.6 mL/min. Five specimens were tested from each composite insulator and the results are presented in Table 2.

In Table 2, the number of the samples that successfully passed the test are presented for each insulator, as well as the average thickness range of the specimens. Half sheds, which were used as specimens for this test, are thinner at the exterior side and thicker at the interior side (close to the glass fiber reinforced plastic rod). Average thickness ranges of the test pieces are also presented in Table 2. Additionally, the average values of maximum depth of erosion for each insulator and the corresponding standard deviations are presented.

From Table 2, it is obvious that only one specimen of C2 insulator failed in this test, due to a hole, which was created on the material during the test. Therefore, the result of the inclined plane test was unsuccessful for this insulator, as the standard [15] requires all five specimens to pass the test in order to indicate the result as “successful”. All the other samples of composite insulators passed successfully the inclined plane test.

From the results of Table 2 and of previous researches, it could be used as a guideline that when the housing material of composite insulators is a high quality HTV silicone rubber with an appropriate concentration of the ATH filler, the result of the inclined plane test must be successful in the most intense voltage level (4.5 kV) [8,16] mentioned in the standard [15], even if the thickness of the specimens does not meet the required 6 mm thickness of IEC 60587 [15]. Furthermore, the maximum depth of the erosion on the HTV silicone rubber with ATH filler material of composite insulators, after the end of the inclined plane test, should be in the range of approximately 0.50 to 0.90 mm, as shown by the results of Table 2.

## 5. Discussion

In this section, a comparative study and a contribution to the field of this research is presented. More specifically, the ranges of the results of the above-mentioned tests on composite insulators, as well as the way of obtaining the specimens for these tests, are presented as an extension of the existing knowledge. It should be mentioned that, in previous researches that contain results for various concentrations of ATH filler in the HTV silicone rubber, the results of approximately 50% by weight concentration were taken into consideration, as this concentration of ATH filler better corresponds to the concentration used for the housing material of composite insulators (finished products). The discussion and comparative analysis are presented below for each test.

### 5.1. Shore A Hardness Measurement

Regarding the shore A hardness measurements on composite insulators, in Table 3 some results from other investigations [8,20,21,22,27] are presented as well as the results of this paper. More specifically, Table 3 includes information about the range of shore A hardness measurement on new (unused–unaged) HTV silicone rubber with ATH filler, which is used as composite insulators’ housing material. A remarkable comment is that in some investigations, the type of test pieces used for the hardness measurement is not mentioned [27]. Hence, the results of these studies cannot be compared with the ones of other studies.

### 5.2. Tensile Strength and Elongation at Break Test

In Table 4, the results of tensile strength and elongation at break from previous investigations, as well as from this research, are presented.

The tested material of all researches was new (unused–unaged) HTV silicone rubber with ATH filler used as housing material on composite insulators. In [4,6,18,19,20], the type of specimens used for the tests is not mentioned by the authors and therefore the results are not comparable. Comparisons should be made on researches that use similar test pieces.

### 5.3. Tear Strength Test

Ranges of the results of tear strength test on new (unused) HTV silicone rubber used as composite insulators’ housing material are presented in Table 5. These results come from other studies, but also from this investigation. It is worthy to mention that most studies in this field do not mention the type of the test piece used for the tear strength test [2,4,19,24]. Thereby, these results cannot be directly compared.

### 5.4. Density Measurement

Regarding the measurement of density of composite insulators’ housing material (HTV silicone rubber with ATH filler), it seems that results of previous studies are similar to those of this study. More specifically, the density of HTV silicone rubber with ATH filler, used as housing material for composite insulators, should be greater than 1.5 gr/cm^3^, as shown in Table 6. This can also be used as an indicator for the quality of the housing material of composite insulator.

### 5.5. Inclined Plane Test

In Table 7, the results of the inclined plane test on HTV silicone rubber with ATH filler material, which is used as housing material for composite insulators, are presented. Table 7 includes results from previous studies [5,8,16,17,20], as well as from this research. From Table 7, it is obvious that when the housing material (HTV silicone rubber with ATH filler) of composite insulators is of good quality, the result of the inclined plane test will be successful, even at the highest test voltage level of IEC 60587, namely, 4.5 kV [15].

## 6. Conclusions

In this paper, five widely used tests in composite insulators’ industry were investigated in detail. More specifically, a methodology for obtaining the specimens from composite insulators (finished products), for each test, is proposed and the ranges of the results for each test are presented. In addition, this research comprises a useful guide for researchers and manufacturers or users of composite insulators who intend to carry out tests in order to develop new materials as composite insulators’ housing and to ascertain the suitability of the insulators’ housing material, respectively. The insulators used in this research were new, unused and unaged medium voltage composite insulators made by six different manufacturers. Housing material of all tested composite insulators in this investigation was HTV silicone rubber with ATH filler, according to manufacturers’ specifications.

In this regard, the main conclusions of this research are the following:

- Some useful analytical methodologies for extracting the test pieces from composite insulators (finished products) for five widely used tests are presented, taking into consideration some deviations, mainly on their dimensions, from the specified ones in the corresponding international standards. As a result, the proposed methodologies could be included to future versions of the used ISO/IEC standards, as extensions.
- The ranges of the results of these tests for new, unused and unaged HTV silicone rubber with ATH filler are presented in this study as an extension and contribution to the existing knowledge. These results in combination with the results of previous investigations, which are also presented in this article, comprise a database that can be used as a guide for future tests on composite insulators’ housing material (HTV silicone rubber with ATH filler) in terms of what the results should be expected to be, so that the material is the appropriate one.
- Shore A hardness measurement on housing material of the available insulators was in the range of 56–75 shore A hardness units. Measurements were taken on one half shed and on the top of two half sheds, layered in opposite direction.
- When measuring on one half shed, with thickness less than the required 6 mm, the result can be multiplied with a correction factor k = 0.99. In this way, the result is “converted” to the measurement on the top of two half sheds, layered in opposite direction, which meets the 6 mm requirement of ISO 48-4 [11]. Alternatively, instead of using k as a correction factor, a factor of 1% could be included in the uncertainty estimation of shore A hardness measurement, when measuring on one half shed.
- Tensile strength test results were in the range of 2.4–6.0 N/mm^2^. Type 2 dumb-bell test pieces [12] were used, which were cut from the sheds of the insulators.
- Elongation at break results were in the range of 150–560%. Test pieces were of the type 2 dumb-bell [12], cut from the sheds of the available composite insulators.
- Tear strength test results range was 12.5–20.0 N/mm. Test pieces were crescent-type [13], cut from the sheds of the insulators.
- Density measurements on the housing material of the tested insulators was in the range of 1.5–1.6 g/cm^3^. Method A of standard ISO 2781 [14] was used and specimens were taken from the sheds of insulators.
- Inclined plane test was successful for 49 out of the 50 tested samples of the available insulators. Test pieces were half sheds and method 1 (constant ac voltage of 4.5 kV for 6 h and 0.6 mL/min flow rate of the contaminant) of standard IEC 60587 [15] was used.
- According to a comparative study with previous investigations [2,4,5,6,8,16,17,18,19,20,21,22,24,26,27], variations of the results of the above-mentioned tests were revealed. These variations depend, among others, on the type of the test pieces, which are used for the tests. It is strongly recommended that the type of test pieces be mentioned in every test report. In this way, comparisons can be made among results obtained from the same test pieces.

As future work in this area, it is proposed that the tests mentioned in this article could be carried out on the housing material (HTV silicone rubber with ATH filler) of composite insulators of even more manufacturers, with different types of test pieces. In this manner, these new results could be presented in comparison/combination with the results of the “Discussion” section of this article. In this way, they could assist the manufacturers of composite insulators during the manufacturing process and the electrical network operators (customers) during acceptance tests, on what results to expect from these tests, in order to ensure the suitability and reliability of the material, as well as researchers during the laboratory tests and the development of the composite insulators’ housing material. 

## Figures and Tables

**Table 1:** Correction factor (k) for the shore A hardness measurements of each available insulator.

Insulator	A1	A2	B1	B2	C1	C2	D1	D2	E1	F1	Avg.	Std. Dev.
*k*	0.99	1.00	0.98	0.99	0.98	0.99	1.00	1.00	0.98	0.99	0.99	0.01

**Table 2:** Analytical results of inclined plane test for the HTV silicone rubber with ATH filler material of all available composite insulators.

Insulator	Passed Samples/ All Samples	Thickness Range (mm)	Max. Depth of Erosion (mm)		Result	Notes
			Average	Std. Dev.		
A1	5/5	2.82–7.13	0.79	0.18	Pass	-
A2	5/5	2.65–5.92	0.85	0.16	Pass	-
B1	5/5	2.97–4.83	0.62	0.12	Pass	-
B2	5/5	2.95–5.04	0.76	0.25	Pass	-
C1	5/5	2.85–5.37	0.84	0.09	Pass	-
C2	4/5	2.79–5.68	0.77	0.14	Fail	Hole
D1	5/5	3.58–8.15	0.51	0.05	Pass	-
D2	5/5	3.84–7.80	0.69	0.28	Pass	-
E1	5/5	2.73–4.70	0.59	0.09	Pass	-
F1	5/5	2.67–5.27	0.55	0.08	Pass	-

**Table 3:** Comparative study and contribution to the range of shore A hardness measurement on new (unused) HTV silicone rubber with ATH filler used as composite insulators’ housing material and the way of the preparation of the test pieces.

Reference	Range of Shore A Hardness Measurement (Shore A Units)	Test Piece
[8]	Not mentioned	Layered sheds
[20]	61–75	Standard test piece (6 mm thick)
[21]	76.64 (no range)	Rectangular test pieces (2 mm thick)
[22]	73 (no range)	Standard test piece (6 mm thick)
[27]	65–75	Not mentioned
This research	56–75	One half shed or two layered half sheds

**Table 4:** Comparative study and contribution to the range of tensile strength and elongation at break on new (unused) HTV silicone rubber with ATH filler used as composite insulators’ housing material and the corresponding types of test pieces used.

Reference	Range of Tensile Strength (N/mm^2^ = MPa)	Range of Elongation at Break (%)	Test Piece
[4]	3.4 (no range)	321 (no range)	Not mentioned
[6]	2.7 (no range)	124 (no range)	Not mentioned
[18]	≥3.0	-	Dumb-bell (Type: not mentioned), cut from composite insulators’ housing material
[19]	≥3.0	-	Not mentioned, cut from composite insulators’ sheds
[20]	4.0–5.1	150–345	Standard dumb-bell (Type: not mentioned)
[22]	6.6 Mpa (67 kgf/cm^2^) (no range)	198 (no range)	Standard dumb-bell (Type: 1)
This research	2.4–6.0	150–560	Dumb-bell (Type: 2), cut from composite insulators’ sheds

**Table 5:** Comparative study and contribution to the range of tear strength on new (unused) HTV silicone rubber with ATH filler used as composite insulators’ housing material and the corresponding types of test pieces used.

Reference	Range of Tear Strength (N/mm)	Test Piece
[2]	≥6.0	Not mentioned
[4]	9.6 (no range)	Not mentioned
[19]	≥7.0	Not mentioned
[20]	13.0–25.0	Standard angle type without nick
[22]	11.0 kgf/cm ≈ 10.8 N/mm (no range)	Standard angle type without nick
[24]	≥7.0	Not mentioned
[26]	>10.0	Standard angle type without nick
This research	12.5–20.0	Crescent type, cut from composite insulators’ sheds

**Table 6:** Comparative study and contribution to density range of on new (unused) HTV silicone rubber with ATH filler used as composite insulators’ housing material and the corresponding insulators’ regions from which the test pieces were extracted.

Reference	Range of Density (g/cm^3^)	Test Piece
[2]	>1.50	Not mentioned
[8]	>1.50	From composite insulators’ sheds and sheath
[20]	≥1.52	Not mentioned
[22]	1.54 (no range)	20 g of material
This research	1.50–1.60	From composite insulators’ sheds

**Table 7:** Comparative study and contribution to the results of inclined plane test, at the most intense voltage level of IEC 60587 standard (4.5 kV) [15], on new (unused) HTV silicone rubber with ATH filler used as composite insulators’ housing material and the corresponding types of test pieces used.

Reference	Result at 4.5 kV	Test Piece
[5]	Pass	Standard specimens
[8]	Pass	From sheath
[16]	Pass	From sheath
[17]	Pass	Half sheds
[20]	Pass	Standard specimens
This research	Pass (49/50)	Half sheds

## Data Availability

The data presented in this study are available on request from the corresponding author. The data are not publicly available due to privacy.
